# Applying data-driven learning in self-translation of academic discourse: A case study of a Chinese medical student

**DOI:** 10.3389/fpsyg.2023.1071123

**Published:** 2023-02-20

**Authors:** Ying Lyu, Ziman Han

**Affiliations:** ^1^Institute of Corpus Studies and Applications, Shanghai International Studies University, Shanghai, China; ^2^School of Foreign Language Studies, Wenzhou Medical University, Wenzhou, Zhejiang, China

**Keywords:** data-driven learning, self-translation, corpus, abstract translation, medical English

## Abstract

This article reports on an experiment on the use of data-driven learning (DDL) in the revision of self-translation by a Chinese medical student. The think-aloud method is employed to investigate the difficulties the student encountered in self-translation and the effectiveness of DDL in improving the quality of self-translation. Results show that difficulties in the self-translation of medical abstracts are mostly associated with markers of rhetorical moves, terminologies, and conventional academic expressions and that they can be effectively solved by such corpus consultation strategies as checking possible options in bilingual dictionaries, using the most certain keywords to find collocations, and using the most possible accompanying words to find contexts. A comparison of translations before and after the application of DDL reveals that it could help improve translation quality in lexical choices, syntactic structures, and discourse practice. An immediate interview shows that the participant holds a positive attitude toward DDL.

## Introduction

Translation plays an important role in the dissemination of academic discourse (Pisanski Peterlin, 2019). In China, it is common for scholars to translate their articles for international publishing purposes. This is also the case in many non-English-speaking countries. Pérez-Llantada et al. (2011) found that two of 10 Spanish academics use translation as their second language (L2) for writing, translating their own writings into English. However, due to these scholars' limited command of L2 and the linguistic and cultural interference during the transfer from L1 to L2, their translations are sometimes not of good quality and even hard for native English speakers to understand, thus risking the possibility of being rejected. As a result, how to improve the self-translation of academic discourse is an issue that bears practical importance for non-native English-speaking scholars who wish to get published internationally.

As corpora record and reflect authentic language use, many applications of corpora have been found in language learning and teaching. These applications have been both indirect and direct. Indirect application refers to specialists using corpus-derived information to design new dictionaries, textbooks, or other teaching materials, while direct application refers to language learners searching and using corpora themselves. The latter is highlighted by Johns (1990) who uses the term data-driven learning (DDL). Thereafter, a large number of studies have been conducted on the effects of DDL on students' second language competence, especially in writing (Huang, 2014; Tono et al., 2014; Samoudi and Modirkhamene, 2022), vocabulary acquisition (Lee and Lin, 2019; Lee et al., 2020), reading comprehension (Hadley and Charles, 2017), and translation (Gavioli, 2005, p. 114-118; Zanettin, 2009; Giampieri, 2019). Among studies on the DDL approach in translation practice, less attention has been paid to the self-translation of academic discourse. Given the fact that self-translation in academic settings is a fairly common practice (Pinto, 2012), such neglect is surprising, and more research should be done.

This study reports a case study of a Chinese medical student using corpora to revise her translation of a research article abstract following data-driven learning methods. It is a process-oriented study aiming to check how effective DDL is at helping a student solve problems in translation. We also intend to find some strategies for using corpora to improve the translation quality of academic discourse. Specifically, this study is going to answer the following four questions:

1. What difficulties would scholars encounter in the self-translation of academic discourse?
2. What corpus consultation strategies are effective in self-translation?
3. To what extent can DDL improve translation quality?
4. What are learners' attitudes toward corpus-based translation?

## Literature review

### Self-translation of academic discourse

Self-translation used to be regarded as a literary phenomenon, referring to authors translating their own literary works into another language (Popovič, 1976, p. 19). Self-translators are often those who are motivated by their intercultural experience with ambitions to create artistic or literary uniqueness (Hokenson, 2013, p. 40), so they are also called author-translators or rewriters (Bassnett, 2013, p. 13). Many studies have been done on literary self-translation, including studies of certain self-translators and their literary works (e.g., Larkosh, 2006; Li, 2017; Sorvari, 2018) as well as theoretical discussions of self-translations (e.g., Ehrlich, 2009; Wilson, 2009, 2017).

However, far less attention has been paid to the self-translation of academic discourse. The early work on this topic is found in Jung's (2002) study that compares self-translated academic texts with students' translations of the same texts. By focusing on linguistic and discourse features, Jung concluded that self-translators tend to adopt translation strategies not seen in other translators. Another typical text-based analysis was done by Al Zumor (2021), who examined the various linguistic strategies that Arab academics took when translating abstracts of their research articles from English into Arabic and found important cross-linguistic differences between the original texts and their translations. In addition, attention has also been paid to the self-translator's attitudes. Pisanski Peterlin (2019) interviewed nine experienced Slovene scholars about their experiences with and attitudes toward self-translation of academic discourse and found that scholars have mixed opinions on it, with some claiming it to be challenging and time-consuming, while others arguing that it was worthwhile and important.

It is surprising that studies on the self-translation of academic discourse are so limited. These studies are either product-oriented, focusing on linguistic features of the translations, or translator-oriented, focusing on translators' attitudes. The process of self-translation has been largely neglected. Our research attempts to examine what difficulties scholars would encounter in translating their own academic texts and whether DDL would help them produce better translations.

### DDL for writing and translation

Data-driven learning (DDL) was first defined as “the use in the classroom of computer-generated concordances to get students to explore regularities of patterning in the target language” (Johns and King, 1991). It usually involves the indirect use of corpus with concordance lines on handouts provided by teachers. Later, direct corpus use by learners was discussed as a contribution of a corpus-based approach to language teaching (Conrad, 2000). DDL emerged when the prevailing paradigm of communicative language teaching (CLT) was questioned. Since CLT emphasizes fluency over accuracy, many language teachers began to call for more focus on grammatical form, and DDL meets this demand. By providing authentic input and asking learners to discover language rules by themselves, DDL creates rich opportunities for focusing on form, drawing learners' attention to language features in context, and improving accuracy in their language output.

Earlier studies on DDL were basically theoretical, but recent years have witnessed an increasing number of empirical studies on second language learning, especially on L2 writing. There are basically three kinds of studies. The first kind focuses on the effect of DDL on the performance of L2 writing, as measured by quantitatively evaluating fluency, accuracy, and complexity (Luo, 2016; Meunier, 2019); by error correction (Crosthwaite, 2017, 2020; Satake, 2020; Zhu, 2021); or by some specific language features such as connectors (Cresswell, 2007). Generally speaking, the DDL approach proves to be effective for L2 writing, although its benefits are not observed in every aspect. The second kind is mainly on the learners' attitudes toward DDL (Geluso and Yamaguchi, 2014; Lin, 2016; Pérez-Paredes et al., 2019). It is found that most learners are positive toward DDL, claiming that corpus consultation can provide authentic language use in context. However, some participants are frustrated while using corpora because it is time-consuming and hard to come up with language rules by themselves. The third kind, which is inspiring to our current research, is about corpus consultation procedures in L2 writing. Kennedy and Miceli (2001) summarized four steps in corpus investigation, namely, formulating the question, devising a search strategy, observing the examples and selecting relevant ones, and drawing conclusions. Quinn (2015) also described a five-step corpora-reference process for self-revision and found that it can force students to consider unexplored aspects of language and refine their linguistic choices.

While there is much literature on corpus-based L2 writing, corpus-based translation practice by L2 learners has been explored to a lesser extent. Very few studies have been done. Scott (2012) and Gallego-Hernández (2015) made surveys on corpus use habits among translators; Gavioli (2005, p. 114–118) and Giampieri (2019) demonstrated that translation quality improves when translators use corpora as references; Yumuk (2002) and Bernardini (2016) advocated the application of corpora in the translation classroom; and Pastor and Alcina (2009) provided a classification of corpora search techniques for translator training. The last study is important to our current research because it lists a category of corpus query probes for translation purposes, supporting our decision-making in choosing the most effective query probes for novice corpus users.

**Figure 1:**
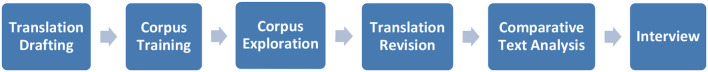
The procedure of DDL-based self-translation of academic discourse.

All these studies give valuable insights into the application of DDL to the translation of academic discourse. First, they confirm that DDL plays a positive role in L2 output (L2 writing and translation). Second, related empirical studies offer useful references on text quality evaluation, corpus user training, and user feedback, helping us design a more reliable research procedure (refer to Figure 1). However, these studies mainly focus on the effects of DDL on translation, and we believe that a thorough investigation of DDL in the translation process is essential because it would expand studies from “why we use DDL” to “how we use DDL” in translation and contribute to both translator training research and DDL research.

## Materials and methods

### Participant

This case study involves a fourth-year undergraduate student majoring in clinical medicine at Wenzhou Medical University, China. She had learned English as a second language for 12 years before entering college and has finished a compulsory 2-year college English course and a 1-year medical English course. She passed China's College English Test (Band 6, CET-6) with a score of 530, indicating that her English proficiency is at an advanced level. In CET-6, the writing and translation tasks are for academic purposes, and her score in these parts proves that she has well-commanded basic academic writing and translating skills. The participant was faced with the task of translating an abstract of her own research article from Chinese into English; she found it difficult and sought support from her English teacher.

Thus, this becomes the task of our experiment: how to translate a medical abstract discussing the effects of a new diabetes treatment in a clinical trial into English. The best way to help her may be to find a specialist who is good at English and familiar with medical academic discourse. Since such a specialist is not always available when needed, a corpus-based data-driven learning (DDL) approach becomes a good alternative. The key point of DDL is that it offers learners a large number of cases of authentic language use, and learners can find specific linguistic features by vertically reading the node(s) in concordance. Repeated patterns can be easily found or judged by their frequency. It is an approach that would assist learners in self-translating abstracts or research articles, at present or in the future, with or without teachers' help.

### Materials

This study experiments with the translation of a Chinese abstract by the participant on diabetes treatment. The Chinese version and its literal translation (by us) are as follows:

Source text in Chinese:

目的:探究新型饮食疗法对2型糖尿病的干预效果。方法:将100例2型糖尿病患者随机分为实验组 (n = 50) 和对照组 (n = 50), 给予对照组患者食物交换份法,给予实验组患者新型饮食疗法。结果:实验组低血糖发生率以及住院时间明显少于对照组 (p ≤ 0.05). 。结论:给予患者新型饮食疗法干预可取得较为满意的效果,值得大力推广应用

Literal translation in English:

*Objective: To find the intervention effects of a new dietary treatment on type 2 diabetes. Methods: 100 cases with type 2 diabetes patients were randomly assigned to a test group (n* = *50) and a control group (n* = *50). The control group was given the food exchange treatment, and the test group was given a new dietary treatment. Results: The rate of hypoglycemia and length of hospital stay of the test group are significantly shorter than the control group (p* ≤ *0.05). Conclusions: The new dieting intervention can achieve relatively satisfactory results, and it is worthy of promotion*.

### Corpus

The corpus used in this study is a self-built monolingual corpus consisting of 60 English research article abstracts from *Diabetes Care*, a leading journal in diabetology, in order to present a closer theme and writing style to our translating task. It would provide authentic language usage in the medical abstract genre in aspects of markers of rhetorical moves (e.g., words like “objective,” “methods,” and “results” in the abstract), terminologies, and conventional academic expressions. The corpus is built by the student herself, under the guidance of the teacher. Before the experiment began, the teacher asked her to download from top-level international medical journals 60 abstracts of articles on diabetes published in recent years. Each abstract was kept as a text file in the txt format. There are 14,438 word tokens in the final corpus. Although not big in size, it serves as a reference corpus with many advantages. First, when compared with large online corpora with millions, even billions, of word tokens, a mini reference corpus can provide language usages more in line with the needs of a specific translation task. The high correlation between input and possible output would make our participants more focused on linguistic forms. Second, because it is a monolingual corpus, it is easy for users to compile. As the participant said, she spent only a few hours building such a corpus. Third, because it is small, it is also quick to retrieve with corpus analysis toolkits. In a word, our goal is to develop a simple, low-cost model for corpus search that the participant can follow in her future translation projects.

### Procedures

Based on Kennedy and Miceli's (2001) four-step corpus investigation process for L2 writing, we made a few modifications and came up with a DDL procedure for a beginner to use corpora for self-translation (refer to Figure 1). It had six steps and was carried out in 4 h, with each step lasting ~40 min. The experiment was done in the teacher's office with only three persons present: the participant, the teacher responsible for instruction, and a research assistant responsible for audio recording and query documentation.

(1) Translation drafting: The participant was asked to translate her Chinese abstract with the help of all sorts of references she can find, either paper/electronic dictionaries or search engines like Baidu. She was asked to report her confusion and uncertainties in translation while drafting with the think-aloud method.
(2) Corpus training: The participant was given a 40-min briefing on corpus use. With the self-made corpus prepared before, we briefed her about Antconc, a freeware corpus analysis toolkit. We first introduced her to basic features in the KWIC concordance interface and then explained the most basic rules of entering search terms. We follow Pastor and Alcina's (ibid.) basic search techniques for translation purposes. The first is probing lexical expressions by a word's lemma or partial form with the combination of a wildcard (^*^) so that all its inflections are displayed in KWIC together. For example, if we want to see how the verb “*investigate*” is used in context, we can enter “investigat^*^” in the search term box and get KWIC results of “*investigate*,” “*investigates*,” “*investigating*,” and “*investigated*.” The second is probing lexical expressions by combining two lemmas with a wildcard (^*^) at each end, such as “control^*^ group^*^” for possible concordance lines of “*control group*,” “*controlling group*,” “*control groups*,” *and “controlling groups*.” It could help the participant confirm her assumption of collocations with grammatical forms.
(3) Corpus exploration: The participant began to try her concordance exploration by trying different search terms with the think-aloud method. Since it was her first time using Antconc, the teacher observed her research process and gave some hints only when she found no clues in concordance research or stopped her think-aloud report for more than 10 s.
(4) Translation revision: Based on the first translated version before the use of corpora, now the participant is asked to make revisions. She was also asked to report why she decided to make such changes to the think-aloud method.
(5) Comparative analysis of texts: The two versions were compared on a qualitative basis to see whether there were any improvements in the second version and what were the limitations of concordance use.
(6) Interview: The participant was asked to report her own attitudes on corpus-based translation revision activity.

### Methods

(1) Think-aloud method

This method was used in translation drafting, corpus exploration, and translation revision. The think-aloud method refers to the method of asking participants to think out loud while performing a given task or immediately after the completion of the task (Eccles and Arsal, 2017). The former is called concurrent reporting, and the latter is called immediate retrospective reporting; our study adopts the former.

The key to concurrent reporting is to involve participants in a specific task and ask them to verbalize all the cognitive processes in a way of speaking to themselves. By using concurrent reporting, researchers can get a picture of what was going on in participants' cognitive processing of a task. In our case, the participant received a 5-min instruction to concurrently think aloud how she translated a given Chinese sentence into English. She would speak out whatever came to her mind, including general comments on the difficulty of the task, uncertainties of lexical equivalents and syntactic structures, worries about translation quality, and efforts to make improvements. If the participant was silent for more than 5 s during the tasks, she was reminded to keep talking aloud. All her reports are recorded and transcribed by a research assistant.

(2) Manual documentation of students' corpus queries

Documenting students' corpus queries can be done manually by the students themselves (Chambers and O'Sullivan, 2004) or through the collection of computer logs (Crosthwaite et al., 2019). In our case, since the participant was busy performing the think-aloud task, the research assistant did the documenting job manually.

The think-aloud transcription and corpus query records are analyzed together to ascertain the participant's cognitive process and corresponding problem-solving action at the same time.

## Results and discussions

### Difficulties in self-translation of academic discourse

The participant felt confused in translating three kinds of expressions, namely, markers of rhetorical moves, medical terminologies (including technical terms and semi-technical terms), and conventional academic expressions in medical abstracts. She found it particularly challenging to express in English the clinical trial design and results. Table 1 shows the participant's final choice for the confusing expressions in the first translation.

**Table 1:** Expressions the participant felt confused.

**Categories**		**Source text (literal translation)**	**Participant's translation**
Markers of rhetorical moves		目的 (objective)	goal
		方法 (methods)	way
		结果 (results)	result
		结论 (conclusions)	conclusion
Medical terminology	Technical terms	饮食疗法 (dietary treatment)	diet therapy
		二型糖尿病 (type 2 diabetes)	Type II Diabetes Mellitus
	Semi-technical terms	干预效果 (intervention effects)	intervention effects
		实验组 (test group)	Test group
Conventional academic expressions		探究 (to find)	searching
		分为…组 (be assigned to)	dividing…into
		明显少于 (significantly less than)	obviously less than
		满意的效果 (satisfactory effects)	a satisfactory way

The participant expressed her confusion by thinking aloud. As for the markers of rhetorical moves, she said that it was easy to find equivalents in her own English vocabulary, but she was not sure whether the equivalent English expressions are commonly used in English medical discourse or in the abstract genre. For example, when she saw “目的 ” (objective), the first equivalent that came to her mind was “*goal*,” which she learned when she was a primary student by reciting English wordlists, with English and corresponding Chinese at the same line. As for “结论” (conclusion), she said that she was not sure if “*conclusion*” could be used alone, because in her memory “*conclusion*” occurred in the fixed chunk “*in conclusion*.” As for medical terminologies, she said that she would check a dictionary for their equivalents, but the problem was that a Chinese-English dictionary often provides more than one expression, and she was not sure which one is more widely used or more idiomatic. For example, the first result for the search of “二型糖尿病 ” in the Baidu online dictionary is “*Type 2 Diabetes*”; the abbreviation of “二型糖尿病 ” in her medical textbook, however, is “T.2 DM,” so she looked for more translations in the dictionary and chose “*Type II Diabetes Mellitus*,” the one most similar to that in her textbook. As for the conventional academic expressions in medical abstracts, she said that they are easy if translated literally, but she was again not sure if they are accepted in the medical community. For example, in describing how to arrange a clinical trial among different groups, the literal translation for “分组” *(be assigned to)* is “*to divide somebody into different groups*,” but she was not sure if native English speakers would understand this expression, although she joked that Chinese readers could. Moreover, she said that she was not certain about the grammatical form of certain words, being plural or single, verb or noun. In addition, she also worried that her translation must be very informal and not up to international publishing standards.

In summary, difficulties in translating academic discourse are usually found in the rendering of markers of rhetorical moves, terminologies, and conventional academic expressions. The reasons, from our analysis of the participant's think-aloud account, are caused by (1) fixed English-Chinese match in English-Chinese vocabulary lists, traditional and still popular among Chinese EFL learners; (2) confusion of too many options in Chinese-English dictionaries; and (3) L1 negative transfer in literal translation.

### Corpus consultation strategies

With the problems identified above in mind, the participant started corpus exploration by herself. The teacher observed her search actions and gave hints when necessary. We recorded what she did and what she thought. The following shows some of the most revealing parts of her “think-aloud” account.

(1) Exploration for markers of rhetorical moves

**Figure 2:**
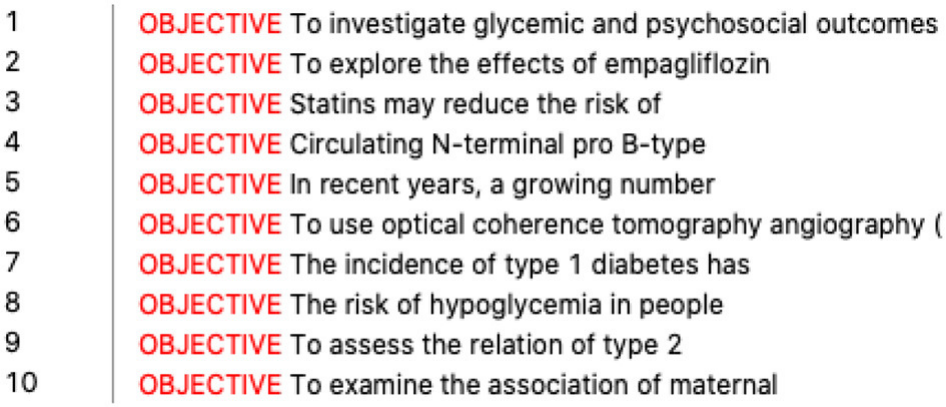
Concordance lines of “objective*”.

First, the student inquired into the marker of the rhetorical move “goal^*^” for translating “目的.” Concordance showed zero hits, which went against her assumption. We suggested she combine the use of electronic dictionaries and corpora if she was not certain about the English equivalent. After checking an electronic dictionary, she found “*target*” and “*objective*” were close to “目的 ” in meaning and decided to check them one by one. The occurrences for “target^*^” were 7, but none served as a marker of a rhetorical move, while “objective^*^” had 64 occurrences, almost all of which were markers of rhetorical moves (refer to Figure 2). The student also realized that “*objective*” as a marker of a rhetorical move is always in the upper case in this journal. Following the same strategy, she found the equivalents for “方法,” “结果,” and “结论” and concluded that the best English translations for the four markers should be “*OBJECTIVE*,” “*METHODS*,” “*RESULTS*,” and “*CONCLUSIONS*.”

(2) Exploration of technical terms

**Figure 3:**
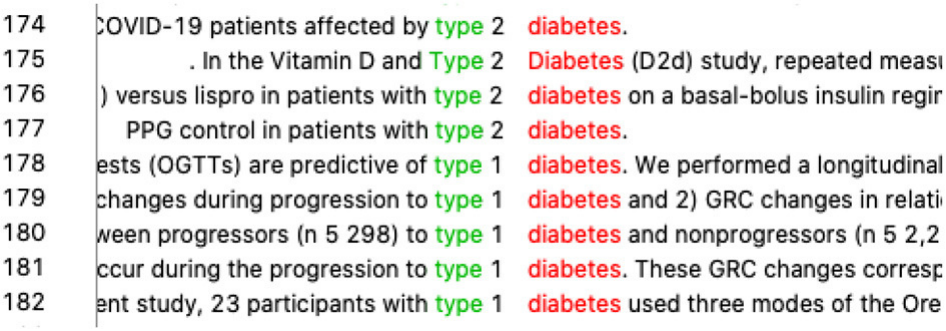
Concordance lines of “diabetes”.

As for technical terms, we suggested our participant first try a keyword that she was most certain about and then find the exact expression for the certain term. For example, when translating “二型糖尿病 ” (type 2 diabetes), the participant did a query of “diabetes^*^” in AntConc and got a list of patterns such as “*type 1 diabetes*” and “*type 2 diabetes*” (refer to Figure 3). She then realized that the English equivalent of “二型糖尿病 ” was in lowercase with Arabic numerals. This conclusion is different from what she found in dictionaries (“*Type 2 Diabetes*” and “*Type II Diabetes Mellitus*”) before.

(3) Exploration of semi-technical terms

**Figure 4:**
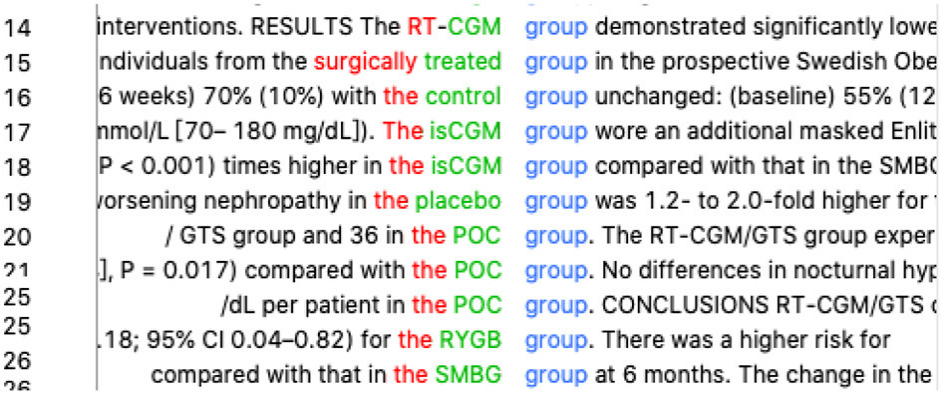
Concordance lines of “group*”.

Translating semi-technical terms is also difficult for the participant because she thought aloud that her translation would be in Chinglish (Chinese English, non-idiomatic English interfered by Chinese features) if they are translated literally. The most typical example is “实验组” (*test group* in literal translation). The participant entered “test^*^ group^*^” in AntConc but found 0 hits, which was against what she had previously anticipated. Since she had successfully found the expression “*type 2 diabetes*” by keying in the most certain keyword “*diabetes*,” a further investigation of “group^*^” (refer to Figure 4) was made to find possible terms for “实验组.” “*Surgically treated group*” was found in the concordance lines, along with several other expressions with capital letters such as “*RT-CGM/GTS group*,” “*POC group*,” and “*RYGB group*.” The student said that she then realized that native speakers use terms of treatments or diseases to name a group in clinical trials, and “*test group*” is not usually accepted. Interestingly, she also felt that “*test group*” may sound inhuman as if some patients are put in a biochemical test, but “*treatment/disease* + *group*” can reduce ambiguity and misunderstanding.

**Figure 5:**
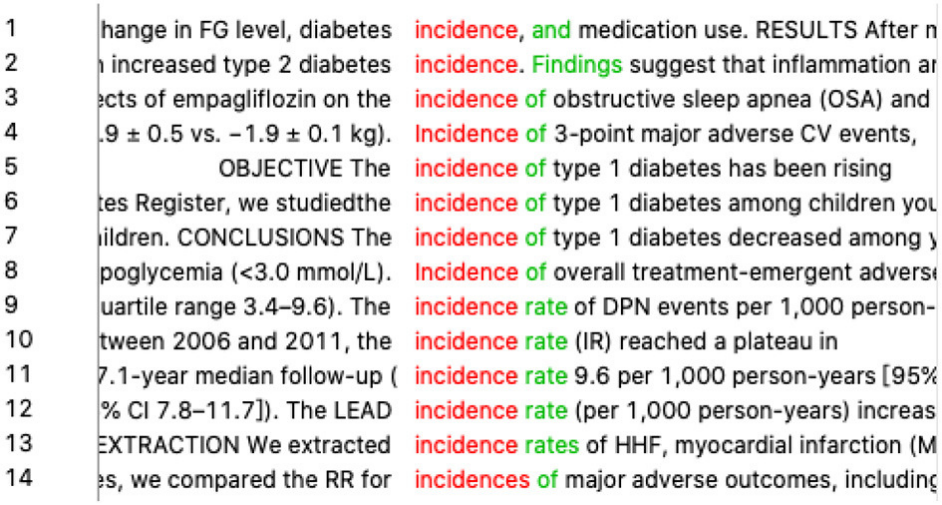
Concordance lines of “incidence*”.

Another example is “发生率” (*happening rate* in literal translation). There are mostly two translations in dictionaries, namely, “*occurrence rate*” and “*incidence rate*.” The student at first wanted to try “*occurrence rate*” directly in the search box, but we told her that a single lemma of “*occurrence*” or “*incidence*” might help find more possible collocations. After searching “occurrence^*^” in AntConc, there was no result, which did not meet the student's expectations. Meanwhile, there were 14 hits for “incidence^*^” (refer to Figure 5), among which five collocated with “*rate*” and nine had no collocates. Thus, the student concluded that “incidence” has the meaning of rate and frequency in itself and that the word “*rate*” was optional.

(4) Exploration of conventional academic expressions

Conventional academic expressions in medical abstracts are usually in the form of fixed syntactic structures. DDL can also play a part in improving translation quality at the sentence level. The strategy we offered is to find the accompanying word, a keyword that often appears in connection with a certain topic in context but not necessarily in a collocation or in the same sentence. For example, in an abstract, if we want to locate expressions for reporting findings of the research, we can look into the context of the accompanying word “*Results*.” Although the marker of the rhetorical move “*Results*” does not appear in the same sentence as the reporting-finding expressions, its context can offer a general picture of lexical choices and syntactic structures.

**Figure 6:**
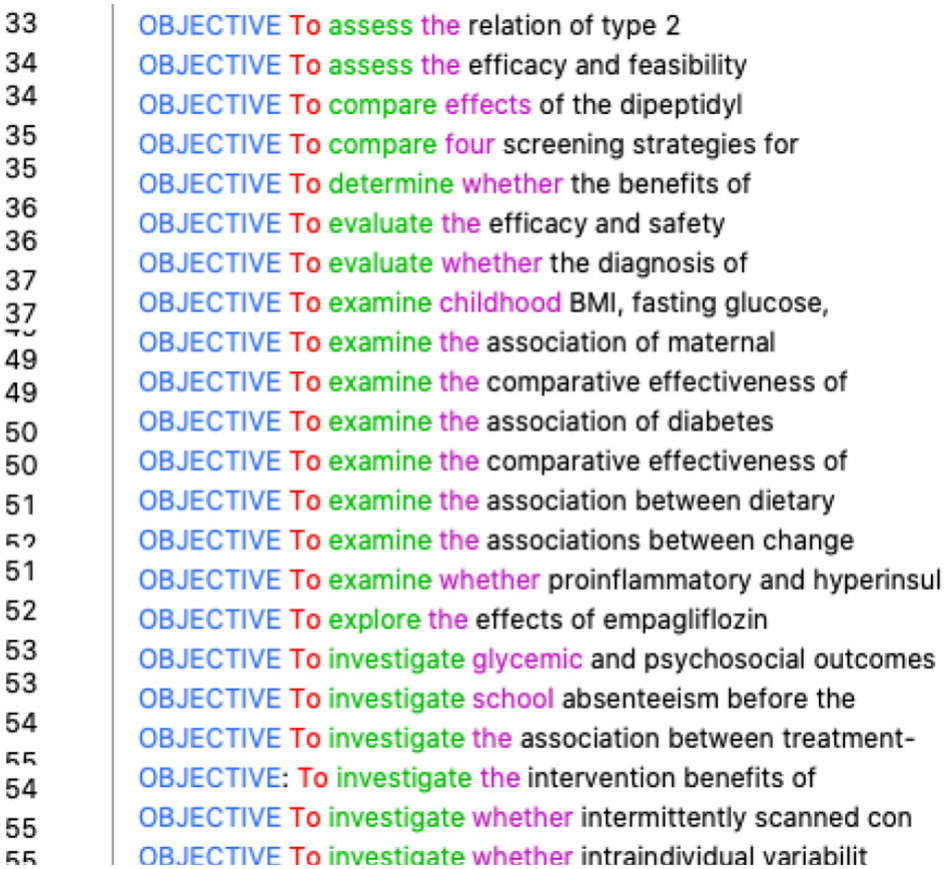
Concordance lines of “objective”.

For example, in translating the first sentence of the abstract introducing the research objective, the participant was not sure how to translate “探究 ” (*search* in the literal translation), not only in lexical choices but also in syntactic structures. We suggested she try its accompanying word. Since the introduction part often occurs after the marker of the rhetorical move “OBJECTIVE,” the student tried “*objective*” (refer to Figure 6) in the concordance and found groups of expressions semantically equivalent to “探究,” such as “*To assess*” (two hits), “*To examine*” (eight hits), “*To evaluate*” (three hits), and “*To investigate*” (six hits). She then realized that the usual English expression for “探究 ” should be a “*TO* + *VERB*” construction with many choices of the verb.

**Figure 7:**
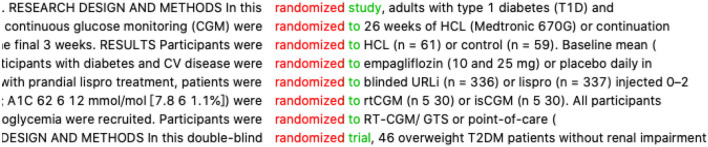
Concordance lines of “random*”.

Next, the student started to explore the conventional expression “分为...组” (*divide … into* in literal translation). As her translation “divide^*^” turned up 0 hits in AntConc, she then tried to identify the accompanying word “*METHODS*.” But this time, the concordance lines are not so clear-cut for her to make a conclusion. Too many methods are involved. At this time, her professional knowledge in medicine gave her some ideas. She said that “分为...组” (*divide … into*) is often preceded by the expression “随机” (*random*) in a clinical trial, so her second accompanying word choice was the English equivalent of “随机.” She consulted the Baidu online dictionary and found “random” to be the closest equivalent. The concordance lines of “random^*^” showed the phrase “randomize(d) to…” in high frequency (refer to Figure 7), and she discovered the syntactic structure “*patients were randomized to A group or B group*” is frequently used.

In summary, the strategies of corpus consultation that prove to be effective in our case are (1) checking possible equivalents offered by bilingual dictionaries; (2) using the most certain keywords to find collocations; and (3) using the most possible accompanying words to find context.

### Comparative text analysis

As mentioned earlier, the evaluation of text quality in L2 writing through DDL is measured by quantitative measurements, such as the general score, fluency, accuracy, complexity, or the number of error corrections. They are not suitable in our case because, on the one hand, our data is small, and on the other hand, the revision of the translation is not error correction, as it is a choice of more acceptable expressions rather than the replacement of wrong ones with the right ones. As a result, we adopted a qualitative approach to analyze key changes between the two translations and then found at least three benefits that DDL can provide in improving translation quality, namely, more accurate lexical choices, more native-like syntactic structures, and more discipline-specific discourse practice.

(1) Lexical choice
Example 1:
*Goal: Searching the intervention effects of a new type of diet therapy for Type II Diabetes Mellitus*. (the first translation)
*OBJECTIVE: To investigate the intervention benefits of a new diet treatment on type 2 diabetes patients*. (the revised translation)

The most obvious changes in the revision are in lexical choices. The marker of rhetorical move “*Goal*” was changed into “*OBJECTIVE*” with all letters capitalized, which was a more common marker of rhetorical move expression in empirical research article abstracts. The verb “*Searching*” was changed into “*To investigate*,” an expression of much higher frequency in concordance, highlighting an action in the future. The noun phrase “*intervention effects*” may include both positive and negative results, and when it was changed into “*intervention benefits*,” its semantic meaning focused only on the positive side, which is what the source text really means. The change of the medical noun phrase “*diet therapy*” into “*diet treatment*” and “*Type II Diabetes Mellitus*” into “*type 2 diabetes*” makes the translation more professional and accurate. We can assume with certainty that corpus-based DDL can provide users with authentic language use and help them confirm the most native-like expressions in lexical choice and their corresponding grammatical form.

(2) Syntactic structure
Example 2:
*Way: Dividing 100 Type II Diabetes Mellitus patients into the test group (n* = *50) and control group (n* =*50), with the control group receiving food exchange therapy and the test group receiving new diet therapy*. (the first translation).
*METHODS: A total of 100 patients with type 2 diabetes were randomized into a food exchange group (n* = *50) and a new diet treatment group (n* = *50)*. (the revised translation)

The key change here is that the verbal structure “*dividing somebody*” in the active voice is replaced by “*somebody be randomized*” in the passive voice. This change is made by concordance proof that native speakers use such expressions to illustrate methods of carrying out a clinical experiment in different groups. When the active voice is replaced by the passive one, the focus of the sentence changes from researchers who conduct the experiment to patients who get involved in the clinical trial, which conforms to the medical discourse where objective facts are more valued than subjective presence. Meanwhile, since our participant found that native speakers use specific treatment or disease names to modify “*group*,” she replaced the expression “*control group is therapied by food exchange method*” with the nominal expression “*food exchange group*,” and “*test group is therapied by new diet therapy*” with “*new diet treatment group*.” When clauses are expressed in nominal phrases, the sentence becomes more concise and clearer.

(3) Discipline-specific discourse practice
Example 3:
*Conclusion: It is a satisfactory way give the patients interventions of new type diet therapy, and it is worth promoting and applicate*. (the first translation)
*Conclusion: The result provides evidence that the new diet treatment can have beneficial effects*. (the revised translation)

The most obvious change here is the deletion of the last sentence in the first version “*and it is worth to promote and applicate*.” This change was made after the participant went through all concordance lines with “*conclusion*” as the accompanying word. She realized that the conclusion section in medical abstracts only reports conclusions in an objective manner, and there is no concordance line about promotion. She thought that it was a shared discourse practice in the international medical community and then boldly deleted the last sentence. It demonstrates that DDL can improve an L2 writer's awareness of discipline discourse conventions and offset the negative influence of L1 linguistic and cultural transfer.

### Learner's attitudes toward DDL

A short interview was conducted immediately to see the participant's attitudes toward corpus-based translation and her difficulties in using the corpus.

The participant's attitude toward DDL was very positive. To her, corpus exploration was an interesting process, and she can check her own assumptions of language use in concordance and find the most native-like expressions. She said that she was very worried about her Chinglish and now she was very happy to find that corpus consultation can help her improve her translation and writing quality in English. She was sure that she would spend more time in the future building a bigger corpus related to her research area and making full use of the corpus in L2 writing and translation.

The participant also thought that the exploration of the corpus was somewhat time-consuming. She remarked that she was not accustomed to reading concordance lines in vertical order. But she thought it was because she had no experience before, and all the difficulties would be overcome if she consulted it on a regular basis.

## Conclusion

This study reports an experiment involving a Chinese medical student using a corpus to improve her self-translation of a medical abstract. We came up with many interesting discoveries.

In translation drafting, we found that difficulties were mostly in looking for equivalents for markers of rhetorical moves, medical terminologies, and conventional academic expressions. According to the participant's think-aloud reports, these difficulties were caused by her fixed English-Chinese match resulting from reciting English-Chinese vocabulary lists, too many options in Chinese-English dictionaries, and negative L1 transfer resulting from literal translations. In corpus exploration, three corpus consultation strategies proved effective in improving translation quality: checking possible equivalents offered by translation dictionaries, using the most certain keywords to find its collocations, and using the most possible accompanying words to find contexts. A comparative analysis of the original translation and the revised version reveals at least three improvements, namely, in lexical choices, syntactic structures, and discipline-specific discourse practice. The participant was very positive about the DDL approach.

Although we made such interesting discoveries, there is still more work we can do in the future. First, based on this pilot study, we can expand our study into different groups of learners with different commands of English and translation skills. We can also do more quantitative text analyses to measure the improvement if the data are big enough. In addition, even though we spend quite a lot of time designing the research, the experiment itself lasts for only 4 h, making the observation of corpus search practice rather limited. As a result, a long-term case study can be followed in the future.

## Data availability statement

The datasets presented in this article are not readily available due to privacy concerns. Requests to access the datasets should be directed at: YL, connie@163.com.

## Author contributions

YL and ZH designed the study. YL collected and analyzed data and wrote the article. ZH supervised the project and revised the article. Both authors contributed to the article and approved the submitted version.

## References

[B1] Al Zumor A. Q. (2021). Exploring intricacies in English passive construction translation in research articles' abstracts by Arab Author-translators. SAGE Open 11, 1–11. 10.1177/21582440211047556

[B2] Bassnett S. (2013). “The self-translator as rewriter,” in Self-translation: Brokering Originality in Hybrid Culture, ed A. Cordingley (London: AandC Black), 13–25.

[B3] Bernardini S. (2016). Discovery learning in the language-for-translation classroom: corpora as learning aids. Cad. Tradução 36, 14–35. 10.5007/2175-7968.2016v36nesp1p14

[B4] Chambers A. O'Sullivan I. (2004). Corpus consultation and advanced learners' writing skills in French. ReCALL 16, 158–172. 10.1017/S0958344004001211

[B5] Conrad S. (2000). Will corpus linguistics revolutionize grammar teaching in the 21st century? TESOL Q. 34, 548–560. 10.2307/3587743

[B6] Cresswell A. (2007). “Getting to ‘know'connectors? Evaluating data-driven learning in a writing skills course,” in Corpora in the Foreign Language Classroom, eds E. Hidalgo, L. Quereda, and J. Santana (Leiden: Brill), 267–287. 10.1163/9789401203906_018

[B7] Crosthwaite P. (2017). Retesting the limits of data-driven learning: feedback and error correction. Comput. Assist. Lang. Learn. 30, 447–473. 10.1080/09588221.2017.1312462

[B8] Crosthwaite P. (2020). Taking DDL online: designing, implementing and evaluating a SPOC on data-driven learning for tertiary L2 writing. Aust. Rev. Appl. Linguist. 43, 169–195. 10.1075/aral.00031.cro

[B9] Crosthwaite P. Wong L. L. Cheung J. (2019). Characterising postgraduate students' corpus query and usage patterns for disciplinary data-driven learning. ReCALL 31, 255–275. 10.1017/S0958344019000077

[B10] Eccles D. W. Arsal G. (2017). The think aloud method: what is it and how do I use it? Qual. Res. Sport Exerc. Health 9, 514–531. 10.1080/2159676X.2017.1331501

[B11] Ehrlich S. (2009). Are self-translators like other translators? Perspect. Stud. Translatol. 17, 243–255. 10.1080/09076760903404050

[B12] Gallego-Hernández D. (2015). The use of corpora as translation resources: a study based on a survey of Spanish professional translators. Perspectives 23, 375–391. 10.1080/0907676X.2014.964269

[B13] Gavioli L. (2005). Exploring Corpora for ESP Learning. Amsterdam: John Benjamins. 10.1075/scl.21

[B14] Geluso J. Yamaguchi A. (2014). Discovering formulaic language through data-driven learning: student attitudes and efficacy. ReCALL 26, 225–242. 10.1017/S0958344014000044

[B15] Giampieri P. (2019). Corpus-based translation in the tourism sector: a case study with final year bachelor students. MediAzioni 24, 1–32. Available online at: http://mediazioni.sitlec.unibo.it

[B16] Hadley G. Charles M. (2017). Enhancing extensive reading with data-driven learning. Lang. Learn. Technol. 21, 131–152. Available online at: http://llt.msu.edu/issues/october2017/hadleycharles.pdf

[B17] Hokenson J. (2013). “History and the self-translator,” in Self-translation: Brokering Originality in Hybrid Culture, ed A. Cordingley (London: AandC Black), 39–60.

[B18] Huang Z. (2014). The effects of paper-based DDL on the acquisition of lexico-grammatical patterns in L2 writing. ReCALL 26, 163–183. 10.1017/S0958344014000020

[B19] Johns T. (1990). From printout to handout: grammar and vocabulary teaching in the context of data-driven learning. CALL Austria 10, 14–34.

[B20] Johns T. King P. (eds) (1991). Classroom Concordancing. Special Issue of ELR Journal 4. Birmingham: ELR.

[B21] Jung V. (2002). English-German Self-Translation of Academic Texts and Its Relevance for Translation Theory and Practice. Frankfurt: Peter Lang.

[B22] Kennedy C. Miceli T. (2001). An evaluation of intermediate students' approaches to corpus investigation. Lan. Learn. Technol. 5, 77–90. Available online at: http://llt.msu.edu/vol5num3/kennedymiceli/

[B23] Larkosh C. (2006). ‘Writing in the foreign' migrant sexuality and translation of the self in manuel puig's later work. Translator 12, 279–299. 10.1080/13556509.2006.10799219

[B24] Lee H. Warschauer M. Lee J. H. (2020). Toward the establishment of a data-driven learning model: role of learner factors in corpus-based second language vocabulary learning. Mod. Lang. J. 104, 345–362. 10.1111/modl.12634

[B25] Lee P. Lin H. (2019). The effect of the inductive and deductive data-driven learning (DDL) on vocabulary acquisition and retention. System 81, 14–25. 10.1016/j.system.2018.12.011

[B26] Li C. (2017). Parallel Corpus-based Study of Literary Self-translation. Beijing: Higher Education Press.

[B27] Lin M. H. (2016). Effects of corpus-aided language learning in the EFL grammar classrooma: a case study of students' learning attitudes and teachers' perceptions in Taiwan. TESOL Q. 50, 871–893. 10.1002/tesq.250

[B28] Luo Q. (2016). The effects of data-driven learning activities on EFL learners' writing development. Springerplus 5, 1–13. 10.1186/s40064-016-2935-5 27536538 PMC4974205

[B29] Meunier F. (2019). “A case for constructive alignment in DDL: rethinking outcomes, practices, and assessment in (data-driven) language learning,” in Data-driven Learning for the Next Generation (New York, NY: Routledge), 13–30. 10.4324/9780429425899-2

[B30] Pastor V. Alcina A. (2009). “Search techniques in corpora for the training of translators,” in Proceedings of the Workshop on Natural Language Processing Methods and Corpora in Translation, Lexicography, and Language Learning, Borovets, 13–20.

[B31] Pérez-Llantada C. Plo R. Ferguson G. R. (2011). “You don't say what you know, only what you can”: the perceptions and practices of senior Spanish academics regarding research dissemination in English. English Specif. Purp. 30, 18–30. 10.1016/j.esp.2010.05.001

[B32] Pérez-Paredes P. Guillamón C. O. Van de Vyver J. Meurice A. Jiménez P. A. Conole G. . (2019). Mobile data-driven language learning: affordances and learners' perception. System 84, 145–159. 10.1016/j.system.2019.06.009

[B33] Pinto A. J. (2012). Reading more intimately: an interrogation of translation studies through self-translation. Salesian J. Humanit. Soc. Sci. 3, 66−72. Available online at: https://ssrn.com/abstract=2136459

[B34] Pisanski Peterlin A. (2019). Self-translation of academic discourse: the attitudes and experiences of authors-translators. Perspect. Stud. Transl. Theory Pract. 27, 846–860. 10.1080/0907676X.2018.1538255

[B35] Popovič A. (1976). Dictionary for the Analysis of Literary Translation. Edmonton: University of Alberta, Department of Comparative Literature.

[B36] Quinn C. (2015). Training L2 writers to reference corpora as a self-correction tool. ELT J. 69, 165–177. 10.1093/elt/ccu062

[B37] Samoudi N. Modirkhamene S. (2022). Concordancing in writing pedagogy and CAF measures of writing. Int. Rev. Appl. Linguist. Lang. Teach. 60, 699–722. 10.1515/iral-2020-2014

[B38] Satake Y. (2020). How error types affect the accuracy of L2 error correction with corpus use. J. Second Lang. Writ. 50, 100757. 10.1016/j.jslw.2020.100757

[B39] Scott J. (2012). “Towards professional uptake of DIY electronic corpora in legal genres,” in Salford Working Papers in Translation and Interpreting. Salford: University of Salford, 1−22.

[B40] Sorvari M. (2018). Altering language, transforming literature: translingualism and literary self-translation in Zinaida Lindén's fiction. Transl. Stud. 11, 158–171. 10.1080/14781700.2017.1399820

[B41] Tono Y. Satake Y. Miura A. (2014). The effects of using corpora on revision tasks in L2 writing with coded error feedback. ReCALL 26, 147–162. 10.1017/S095834401400007X

[B42] Wilson R. (2009). The writer's double: translation, writing, and autobiography. Roman. Stud. 27, 186–198. 10.1179/174581509X455150

[B43] Wilson R. (2017). “Forms of self-translation,” in Reconstructing Identity, eds N. Monk, M. Lindgren, S. McDonald, and S. Pasfield-Neofitou (Cham: Palgrave Macmillan), 157–177.

[B44] Yumuk A. (2002). Letting go of control to the learners: the role of the Internet in promoting a more autonomous view of learning in an academic translation course. Educ. Res. 44, 141–156. 10.1080/00131880210135278

[B45] Zanettin F. (2009). Corpus-based translation activities for language learners. Interpret. Transl. Train. 3, 209–224. 10.1080/1750399X.2009.10798789

[B46] Zhu F. (2021). Supporting EFL writing during the pandemic: the effectiveness of data-driven learning in error correction. Asian EFL J. 25, 8–27. Available online at: https://eprints.lancs.ac.uk/id/eprint/157758

